# Description of Zoonotic Pseudocowpoxvirus Infection of Cattle in Russia

**DOI:** 10.3390/ani14060969

**Published:** 2024-03-20

**Authors:** Irina Sindryakova, Andrey Blokhin, Valentina Lyska, Ilya Titov

**Affiliations:** Federal Research Center for Virology and Microbiology (FRCVM), Academician Bakoulov Street, Bldg. 1, 601125 Volginsky, Russia; and.bloxin2010@yandex.ru (A.B.);

**Keywords:** zoonotic diseases, pseudocowpoxvirus, epidemiology, clinical signs cattle, humans, skin lesions, molecular properties, differential diagnosis

## Abstract

**Simple Summary:**

This work describes a case study of pseudocowpoxvirus (PCPV) infection in Russia (Irkutsk region). The clinical manifestation, molecular identification, and molecular characterization of the disease were established. Epidemiological information were provided by the state veterinary service; additionally, the cows’ owners were interviewed using a semi-structured questionnaire. The description of clinical symptoms was based on clinical examination and farmer interviewing. Samples (blood, serum, scab) were collected from the affected cattle for virological studies and molecular analyses. The identification of PCPV was accomplished through the B2L gene fragment amplification, sequencing, and phylogenetic analysis. Based on the nucleotide identities and phylogenetic analysis of the partial-length B2L gene, the Irkutsk 2019 isolate was classified as PCPV. Because of high rates of virus transmission amongst cattle-to-cattle and cattle-to-humans through direct contact, it is important to raise farmers’ awareness of the clinical signs of the disease, highlight its preventive measures, and to develop a strategy to motivate them to seek qualified veterinary care.

**Abstract:**

Parapoxviruses are worldwide epitheliotropic viruses that affect ruminants. Viruses of this genus have a narrow host range; however, the pseudocowpox virus (PCPV) also infects humans. Unfortunately, these cases are not well documented, and the epidemiology and the properties of the causative agents are not properly described. Here, we report the first case of PCPV in northern Russia (the Irkutsk region). The infection occurred in non-immune herds where no new arrivals of animals had been reported. Moreover, clinical signs of infection (skin lesions) were observed in humans. Based on the nucleotide identity and phylogenetic analysis of the partial-length B2L gene, the Irkutsk 2019 isolate was classified as PCPV. Phylogenetic analysis based on the nucleotide sequence of the B2L gene fragment of PCPV revealed a close phylogenetic relationship between the Irkutsk 2019 isolate and the PCPV strains isolated in Europe and the USA. The high degree of conservatism of the B2L gene does not allow for finding a correlation between their geographical origin and the results of phylogenetic analysis.

## 1. Introduction

Pseudocowpox virus (PCPV) is a member of the *Parapoxvirus* genus within the *Poxviridae* family, encompassing various viruses that induce diseases of significant clinical and economic consequences. Parapoxviruses are epitheliotropic viruses that affect ruminants and cause papular stomatitis and contagious pustular dermatitis in the areas of the lips, nostrils, oral mucosa, and teats [1,2].

The PCPV infection is characterized by sporadic or endemic spread in numerous global regions [3,4,5]. PCPV infection is typical for dairy cows [6] and manifests itself as macules, papules, and pustules with the formation of scabs on the teats [7,8,9]. PPVs are zoonotic and cause skin lesions in humans from direct contact with infected, animals commonly cows and sheep [10]. In addition, PPVs affect other ungulates species, such as red deer [11], reindeer [4], chamois, ibex [12], musk oxen [13], camels [14], gazelles [15], and wild Japanese serows [16]. Infections have also been reported in non-ungulates, such as red squirrels, gray squirrels [17], seals [18], and pygmy chimpanzees [19].

While Russia has not previously reported parapoxvirus infections in animals and humans, this study offers an initial description of PCPV infection in cattle in our country.

This article presents the clinical manifestation, molecular identification, and molecular characterization of the first case of PCPV in eastern Siberia (Irkutsk region, Russia).

## 2. Materials and Methods

**Figure 1:**
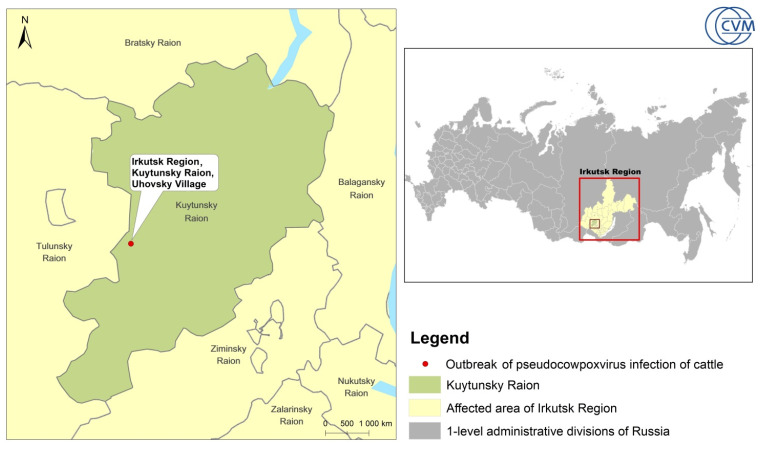
Outbreak and sample collection site.

In September 2019, a total of 16 dairy cows (Holstein Friesian breed) on a farm in Ukhovsky village, Kuytun district, Irkutsk region (E 101.136976: N 54.403191) (Figure 1), developed skin lesions on their teats.

Cattle production in the district is mainly presented by small farms (backyards) that keep 2–3 animals for self-consumption. In addition, the village keeps sheep and goats, usually together with cattle.

The owners practiced communal and mixed grazing from June to September.

To describe the situation and course of the disease, a retrospective epidemiological analysis was performed. Epidemiological data were provided by the state veterinary service of the Irkutsk region and Kuytun district. Additionally, the owners were interviewed using a semi-structured questionnaire. The questionnaire was designed to assess whether the informants know PCPV infection, its clinical signs, source of infection, prevention, and control measures. When administering the questionnaire, the interviewer explained to the respondents by whom and for what purpose this study was being conducted. The participants were also informed that the information they provide about themselves is strictly confidential. People who did not wish to be voluntarily interviewed could refuse. The survey was not initiated until the informed consent was signed. The questionnaire included open-ended questions to determine the owners’ awareness of the disease, its clinical signs, prevention, and treatment measures, as well as possible sources of the disease, presence of animals with similar symptoms in the last 2 years, time of detection of the first sick animals, and veterinarians’ prescribed treatment or self-treatment of sick animals.

The description of clinical symptoms was based on clinical examination and farmer interviewing.

### 2.1. Sample Collection

Nine samples (blood, serum, scab) were collected from the affected cattle for virological studies and molecular analyses. Samples were collected from sick animals by skilled veterinarians: blood specimens were collected in test tubes (5 mL each with EDTA for virus genome detection. Scabs transported in special transport media in polypropylene tubes.

### 2.2. Molecular Diagnostics and Analysis

In the laboratory, the scab samples were mechanically homogenized with phosphate-buffered saline (PBS). The viral DNA was extracted by using a QIAamp_DNA Mini kit (QIAGEN GmbH - Hilden, Germany) according to the manufacturer’s instructions.

The isolated DNA was examined to identify eight poxviruses of medical and veterinary importance [20], and of importance for viral infections causing exanthematous and vesiculo-papular skin lesions of the udder and teats of dairy cows [21].

For the amplification and sequencing of partial-length (374 bp) B2L gene encoding the major envelope protein of parapoxvirus, the primer set PCPVF/PPVR [21] was used.

The cycling conditions were composed of 3 min at 94 °C, followed by 40 cycles with 94 °C for 15 s, 50 °C for 30 s, 72 °C for 45 s, and a final extension cycle of 72 °C for 5 min.

The amplicons were visualized in 1.5% TAE gel.

PCR products from the gel were purified with a QIAquick Gel Extraction Kit (QIAGEN GmbH - Hilden Germany). A nucleotide sequence was determined by a direct sequencing method using a BigDye Terminator Cycle Sequencing Kit v3.1 (Applied Biosystems, Austin, TX, USA). Sequence data were aligned by using the ClustalW method [22]. Phylogenetic analysis was performed by using MEGA X software, version 10.2.5 [23].

Phylogenetic trees were constructed using the maximum likelihood method, utilizing the Tamura–Nei model. The reliability of the branches was evaluated by using the bootstrapping method with 1000 replicates. Nucleotide sequences were compared with the sequences of corresponding parapoxviruses available in GenBank.

### 2.3. Virus Isolation

Due to the lack of primary bovine testis (BT) cell culture, we used cow lung embryo cells and Madin–Darby bovine kidney (MDBK) cell culture from the FRCVM cell culture bank to isolate the virus. The samples (scabs) were suspended in 10% Eagle’s MEM medium (Sigma-Aldrich, St. Louis, MO, USA) with the addition of antibiotics (500 U/mL of penicillin, 250 μg/mL of streptomycin, and 60 U/mL nystatin). Then, 1.0 cm^3^ of this suspension was transferred into a culture flask (surface area 25 cm^2^). After one hour of exposition (adsorption), the suspension was removed and a maintenance medium containing 2% bovine serum was added. The flasks were incubated at 37 ± 0.5 °C for 5 to 6 days and examined for cytopathic effects (CPE) daily.

## 3. Results

### 3.1. Epidemiology

The disease was registered in a village with a population of 1256 people. Furthermore, 122 heads of cattle were kept in 32 backyards in the village.

The survey revealed that 52.0% (28/54) of the surveyed farmers had no information on the PCPV infection but knew its various names. Those who knew about the disease were instructed to indicate the typical symptoms of the disease. All the farmers who know about the disease unequivocally described one clinical symptom: scabs on the teats of cows’ udders. At the same time, the formation of roseolae, nodules, and pustules was not considered as a sign of any disease by the farmers. None of the interviewees had encountered similar clinical signs in heifers and steers. Additionally, 64.3% (18/28) of the farmers believed that milking led to PCPV infection, while about 43.0% (12/28) attributed the outbreaks to the dirty hands of the milkers. However, 87.0% (47/54) of the interviewed cattle owners did not consult veterinarians for treatment of animals. All the animal owners were not aware of PCPV infection prevention measures.

The survey results revealed that 35.2% (19/54) of the surveyed cattle owners reported having cases of PCPV infections in cattle in their village in the previous 2 years. None of the farmers had sought qualified veterinary care, considering the disease to be non-threatening.

### 3.2. Clinical Signs

**Figure 2:**
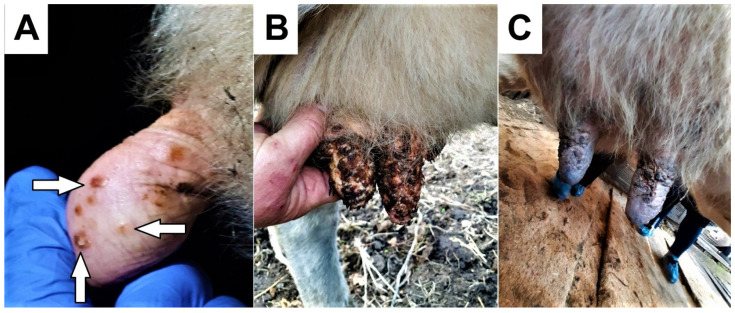
Lesions on the tits of a dairy cow at the age of 4 years: (**A**) The formation of macula (1st day) and papules (3rd day). (**B**) The formation of scabs (5th day). (**C**) The beginning of the regenerative process (8th day).

Initially, a round or oblong redness (roseola) appeared on the nipple, which lasted from 12 h to a day. This was followed by the formation of a dense nodule, which turned into a pustule within 2–3 days. The pustule took the form of a bubble with a red rim and an indentation in the center. After the pustule ruptured, a scab covered with a dark crust formed. The duration of the disease from the appearance of clinical signs to recovery ranged from 7 to 14 days, with most animals recovering within a week. Treatment was administered by the owners using veterinary ointments, creams, and antibiotics. All animals recovered without scar formation (Figure 2).

Examination of a young bull and calves did not reveal any signs of skin damage on the scrotum, udder, or oral mucosa.

**Figure 3:**
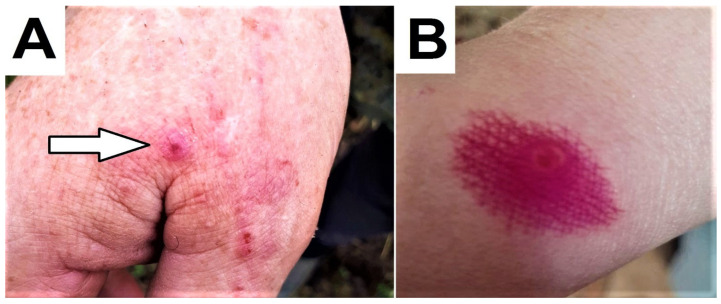
Damage to the skin of the milker’s hands who came into contact with animals during manual milking. (**A**) Macula (1st day). (**B**) Pustule formation (4th day).

At the same time as the animals’ illness, their handlers, who practiced manual milking, also developed skin lesions on their hands (Figure 3). These manifested as the formation of macules and roseola, which turned into pustules. Dermal wound healing occurred without scar formation.

### 3.3. Molecular Genetic Analysis

**Figure 4:**
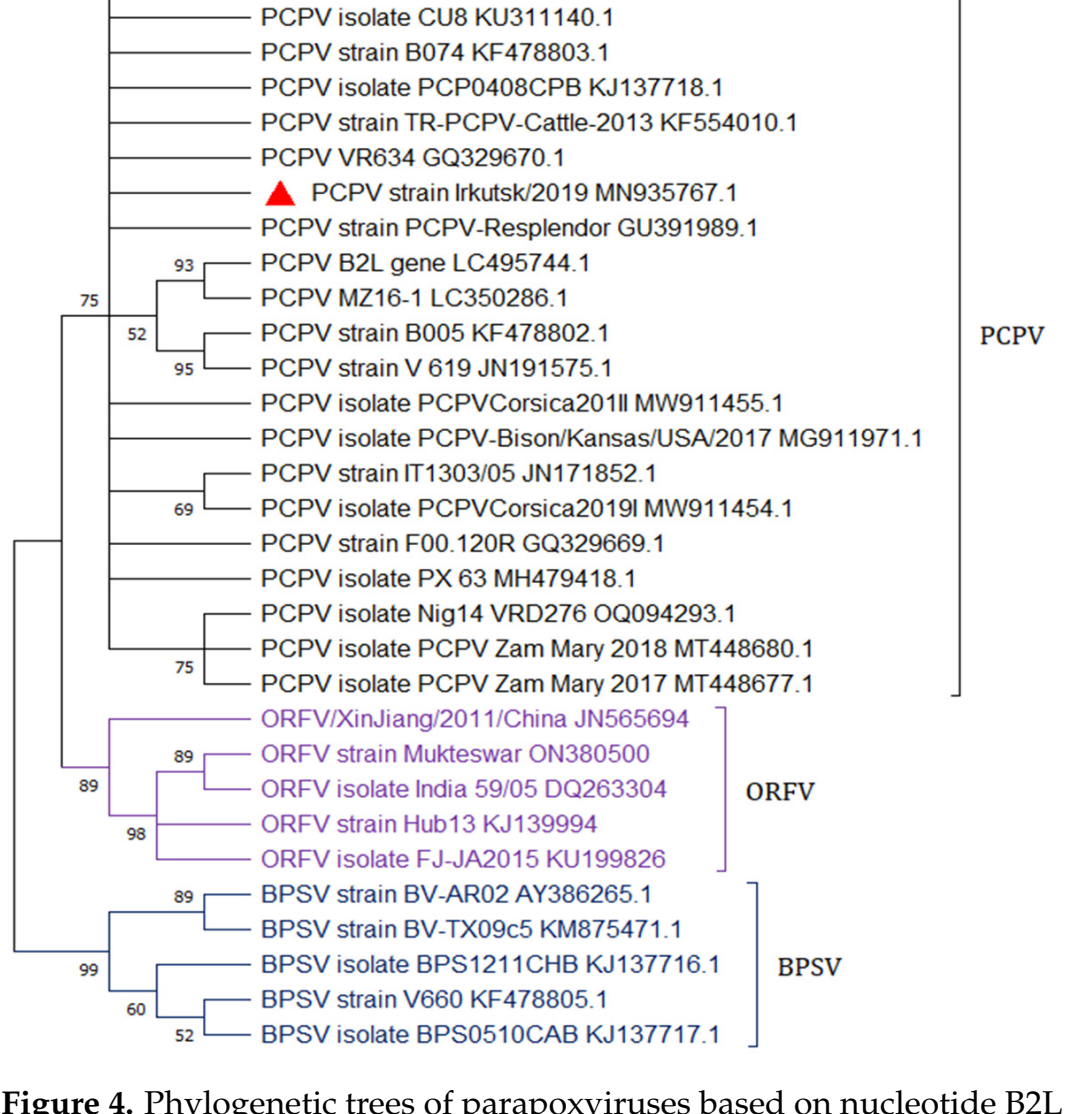
Phylogenetic trees of parapoxviruses based on nucleotide B2L gene fragment sequences. The percentage bootstrap values calculated from 1000 replications are indicated above the internal nodes. This analysis involved 31 nucleotide sequences. The codon positions included were 1st + 2nd + 3rd + non-coding. Russian isolate Irkutsk/2019 is highlighted with a red triangle.

Molecular genetic analysis of the samples showed that the disease was caused by a representative of the pseudocowpoxvirus family. The identification of PCPV has been accomplished through B2L gene fragment amplification, sequencing, and phylogenetic analysis [11,24,25,26]. It showed a high degree of homology between the sequences deposited in GenBank. The nucleotide identities ranged from 96.79 to 99.73%. Based on the nucleotide identities and phylogenetic analysis of the partial-length B2L gene, the Irkutsk 2019 isolate (GenBank accession number MN935767) was classified as PCPV (Figure 4).

### 3.4. Virus Isolation

**Figure 5:**
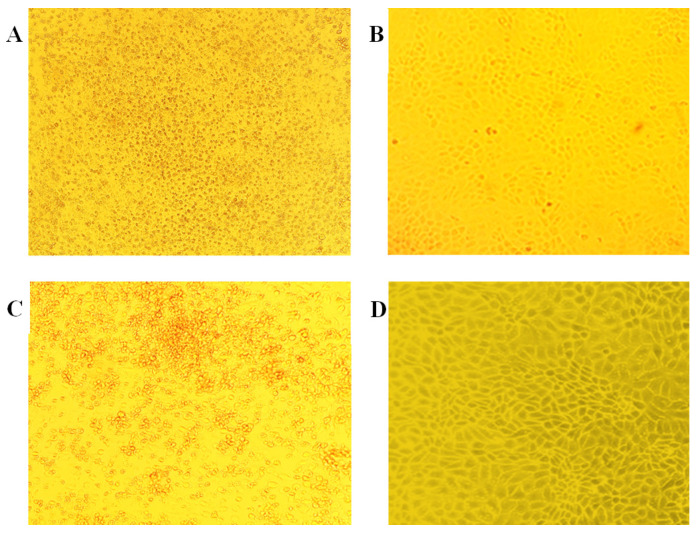
MDBK and bovine lung embryonic cells monolayers after inoculation of the viral material. (**A**) Bovine lung embryonic cell culture with 70% of CPE. (**B**) Control bovine lung embryonic cell culture. (**C**) MDBK cell culture with 50% of CPE. (**D**) MDBK control cell culture.

In parallel with the genetic studies, virus isolation in cell cultures was carried out. Usually, CPE is observed in cell cultures at 1–4 days post-infection and is characterized by enlarged cytoplasm, rounding, and full cell detachment. In this study, after the expiry of a 24 h post-inoculation period of the viral material, 50% of MDBK cell culture and 70% of bovine lung embryonic cells had CPE, which was characterized by round morphology and cell detachment (Figure 5A,C, respectively). However, at the second passage, only 10% CPE was observed in both cell cultures, and at the third passage, CPE was not present at all (Figure 5).

PCR was used as a confirmatory protocol for virus isolation at each passage of cell cultures. From the first two passages, positive PCR results were obtained, which were indicated by the presence of specific bands in the agarose gel. The PCR results of the sample from the third passage were negative, which indicated an absence of viral nucleic acid and virus replication.

## 4. Discussion

Although PCPV in cattle is known to be prevalent worldwide and cause financial losses in livestock production [3,25,27], there have been no official reports of the disease in Russia. The low incidence of parapoxvirus infection in cattle is likely due to the asymptomatic nature of the infection and its latent persistence [28]. Therefore, this is the first identification of PCPV in Russia.

Parapoxiviruses exhibit a limited host range; however, PCPV induces dermatitis in humans [6]. PCPV is commonly referred to as an “occupational disease” among milkers and support staff, resulting in localized skin lesions known as “milker’s nodules” or pseudocowpox [5]. While PPV infections in humans are uncommon, individuals at higher risk, such as slaughterhouse workers, veterinarians, farmers, and animal keepers, have an increased likelihood of contracting the virus through direct contact with the infectious source [29,30]. Additionally, infection can occur through visits to contact zoos or through occupational exposure to furs, skins, or meat contaminated with the virus [31,32]. Furthermore, a correlation has been reported between a widespread human pox infection and the ritual sacrifice of sheep during the Islamic holiday “Feast of Sacrifice” [33,34].

The virus is excreted by infected animals that are introduced without undergoing quarantine and spreads slowly within a herd. Transmission within the herd occurs through direct contact, such as a calf sucking on multiple cows, as well as indirect contact through flies, milking equipment, and the hands of milkers. The virus enters the body through skin damage or sometimes through the oral mucosa, where it then replicates in keratinocytes [3].

Our field investigations demonstrate that the skin lesions were observed only on the teats and udder in 13.1% of the cows in the herd. There were no lesions on other animal body parts, including the limbs, mouthparts, ventrum, and skin folds. This is typical for a mild form of the disease. But despite the mild form of the disease in cows, the virus had significant zoonotic potential, which was manifested by its infection of the hands of milkers who practiced manual milking. Other people did not show the described lesions or deterioration in health. Thus, the virus that caused the disease in cows had a weak zoonotic potential, which could only be realized through direct contact during milking.

The timing and method of introduction of the PCPV into the herd remains undetermined. The owner reported no apparent signs of PCPV infection prior to the outbreak.

The current situation with PPV infection in cattle in Russia is unclear because it rarely becomes widespread, there are no rapid and accurate diagnostic tools, and it is not subject to mandatory notification. The case we describe here attracted attention due to the large number of sick animals. With the involvement of veterinarians, the outbreak was quickly contained using antiviral drugs and antiseptic ointments. Livestock premises and milking equipment were also disinfected. At the same time, differential diagnostics were carried out for infections such as foot and mouth disease, bovine viral diarrhea, epizootic hemorrhagic disease, bluetongue, ovine herpesvirus 2, and bovine herpesvirus 1.

Molecular genetic analysis revealed that the causative agent of the disease in animals was pseudocowpox virus [3,35].

We have conducted a comparative analysis of our sequence with Orf virus, PCPV, and BPSV obtained from various animal and human sources present in the GenBank database. The results of this comparison were as follows: the highest identity (99.73%) was found compared to five PCPV strains with the following GenBank accession numbers: KF554010 (strain TR-PCPV-Cattle-2013) and JN171856 (strain 1/07), isolated from cattle in 2013 in Turkey and in 2012 in Germany, respectively; MH479410 (isolate PX 104, Georgia, 2018), KF478803 (strain B074, Germany), and GQ329670 (strain VR634, USA, 1963), isolated from the hands of “milker nodes” on the hands of affected people (Figure 4).

The source of the virus’s introduction to farms has not been identified. However, there is a documented a case of PCPV infection in a cat, which was transmitted from a cow [36]. The interspecies transmission of PCPV can lead to genetic mutations, enabling the virus to infect other animals and potentially humans [37]. Consequently, these findings have broadened the host range of PCPV and imply that novel mutated strains could be identified in animal species that were previously unaffected by PCPV infections. Once the species barrier is breached, the emergence of new mutant viruses becomes plausible, increasing the likelihood of human-to-human and/or animal-to-animal transmission [38].

Zoonotic PCPV has been identified in the cattle population of Russia, and it is crucial to include this pathogen in the diagnosis of vesicular diseases in both bovines and humans.

## 5. Conclusions

The present study describes the first detection and clinical and epizootological characterization of PCPV in Russia, which demonstrates the presence of the disease in a cattle population. However, there is a need to raise farmers’ awareness of the clinical signs of the disease, to highlight its preventive measures, and to develop a strategy to motivate them to seek qualified veterinary care. The correct identification of the causative agent of bovine pox-like diseases is essential for proper veterinary intervention, including rapid and quality diagnosis and treatment of animals. Similarly, when PCPV infection is identified, strict hygienic measures are necessary to prevent its transmission to humans. A nationwide surveillance campaign is needed to assess the prevalence and spatial distribution of PCPV, as well as to identify risks of the disease spreading in Russia.

## Data Availability

The dataset presented in this study can be found in online repositories. The name of the repository and accession number can be found at: https://www.ncbi.nlm.nih.gov/genbank/, MN935767.1 (accessed on 3 November 2020).
